# Do malignant cells sleep at night?

**DOI:** 10.1186/s13059-020-02179-w

**Published:** 2020-11-12

**Authors:** Luis Enrique Cortés-Hernández, Zahra Eslami-S, Antoine M. Dujon, Mathieu Giraudeau, Beata Ujvari, Frédéric Thomas, Catherine Alix-Panabières

**Affiliations:** 1 Laboratory of Rare Human Circulating Cells (LCCRH), University Medical Centre of Montpellier, Montpellier, France; 2 CREEC (CREES), Unité Mixte de Recherches, IRD 224–CNRS 5290–Université de Montpellier, Montpellier, France; 3 Centre for Integrative Ecology, School of Life and Environmental Sciences, Deakin University, Waurn Ponds, Victoria Australia; 4 School of Natural Sciences, University of Tasmania, Hobart, Tasmania Australia; 5 Institut Universitaire de Recherche Clinique (IURC), 641, avenue du Doyen Gaston Giraud, 34093 Montpellier Cedex 5, France

**Keywords:** Circadian cycle, Tumor dissemination, Circulating tumor cells, Chronobiology, Phenology, Disease ecology

## Abstract

Biological rhythms regulate the biology of most, if not all living creatures, from whole organisms to their constitutive cells, their microbiota, and also parasites. Here, we present the hypothesis that internal and external ecological variations induced by biological cycles also influence or are exploited by cancer cells, especially by circulating tumor cells, the key players in the metastatic cascade. We then discuss the possible clinical implications of the effect of biological cycles on cancer progression, and how they could be exploited to improve and standardize methods used in the liquid biopsy field.

## Introduction

Since the early 1940s, multicellular organisms are no longer considered autonomous entities, but rather “holobionts,” i.e., assemblages composed of the host and its associated commensal and mutualistic microorganisms and parasitic taxa [1, 2]. Recently, Thomas et al. [3] proposed that multicellular organisms have a long evolutionary history with a third category of living entities inside their bodies: cancer cell communities (oncobiota). From precancerous lesions to metastatic cancers, oncogenic processes are very frequent in humans and animals [4, 5], and not just during aging as often thought in the past [6]. From an evolutionary perspective, the transformation of normal cells into malignant cells that will form a cancer is equivalent to a speciation event inside the body [7, 8], preceded by a set of mutations that allow normal cells to acquire self-defined fitness functions and on which natural selection can act [9].

As the holobiont components are usually prisoners of their host, their environment is composed of at least two very different ecological dimensions: the host (i.e., the immediate environment) and the host habitat (i.e., the ecosystem). The first type of environmental variables that holobiont members experience are the host physiological, genetic, and phenotypic characteristics, such as sex, age, and immunocompetence. The second level of environmental variability is due to the biotic and abiotic factors that characterize the ecosystem in which the host lives (resource level, abundance of predators, seasons). These intra- and inter-individual variables and their interactions contribute to the selective landscape in which holobiont members survive and maximize their lifetime reproductive success (i.e., their fitness). Examples of host-parasite interactions show that several parasite species have adapted to perceive, and then to respond in a state-dependent manner, to various signals linked to immediate and/or external fitness-related environmental parameters [10].

An interesting direction in this research area concerns the influence of the biological rhythms that coordinate organismal activities with the environment circadian rhythms. Importantly, holobiont members are not only influenced by the rhythm of the external biotic and abiotic environments, but also of the host biotic environment. Several studies have demonstrated the relevance of these biological cycles in host-parasite relationships. For example, parasites manifest periodic modulations of traits, such as virulence, development, and transmission, in function of their host behavioral or physiological rhythms, such as periodicity in the immune responses and/or host feeding rhythms [11–14]. Hosts may use biological rhythms to defend themselves against parasites (e.g., circadian clocks in immune cells can modulate the magnitude of *Leishmania* infection) [15], but parasites also may manipulate the host clocks to their own advantage. For instance, baculoviruses called *Spodoptera exigua* multiple nucleopolyhedroviruses (SeMNPV) can capture their host phototaxis pathway, thus forcing the host to climb to elevated positions that are more favorable for parasite transmission [16, 17]. Similar reciprocal interactions also exist between the host and its microbiota. Indeed, the microbiota rhythms and consequently their community structure and metabolic activity are regulated by their host diet and feeding time [18]. On the other hand, intestinal microbiota can, sometimes, program the host diurnal metabolic rhythms, thus influencing diurnal fluctuations of the host physiology and susceptibility to pathologies [19].

**Fig. 1:**
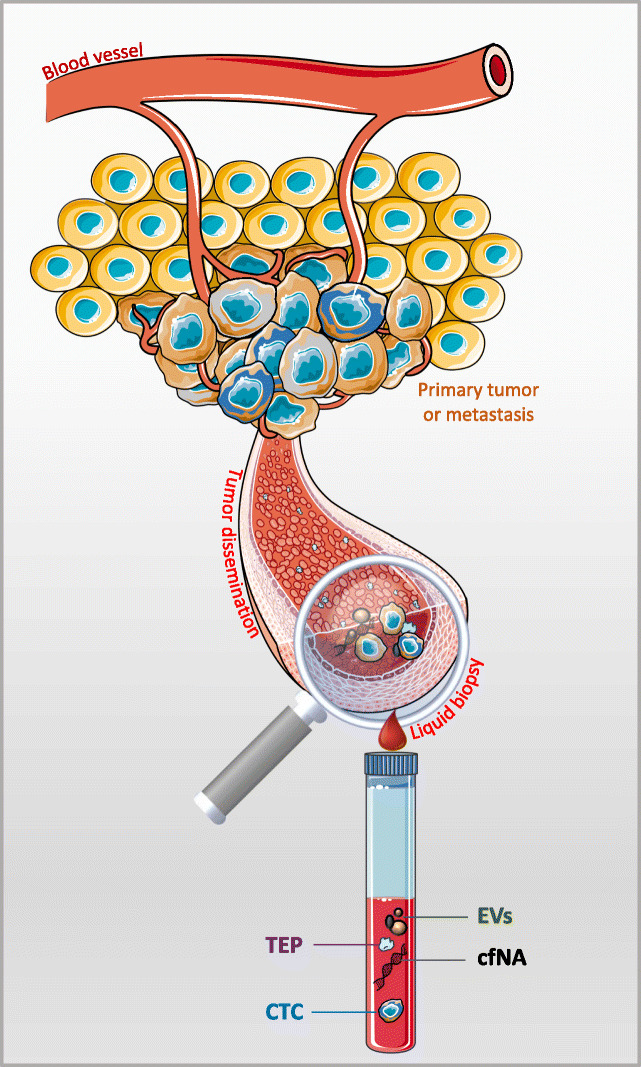
Liquid biopsy. In cancer, the liquid biopsy term describes the minimally invasive analysis of analytes released by or related to the primary and/or metastatic tumors. These analytes can be found in any physiological or pathological body liquid (e.g., blood, ascites) [20]. This is an extension of the tissue biopsy, and many cancer biomarkers of clinical utility can be found also in liquid biopsy samples. Moreover, new biomarkers can be easily identified in liquid biopsy analytes because they are thought to represent more accurately cancer progression (i.e., the metastatic cascade), and cancer heterogeneity than tissue biopsy samples [21]. Some examples of liquid biopsy analytes are: circulating tumor cells (CTCs), circulating-free nucleic acids (cfNA: DNA or RNA), extracellular vesicles (EVs), tumor-educated platelets, and their possible combination with other protein tumor makers [22]. Although all these analytes have biological significance during the metastatic cascade and provide useful clinical information, currently, the most studied analytes are CTCs and cfNA. As CTCs are the main drivers of the metastatic cascade, it is reasonable to suggest that the biological cycles might influence their biological behavior, as observed in cancer. Consequently, these observations have implications for the current and future clinical applications of CTCs as liquid biopsy

The connections between chronobiology, disease ecology, and evolutionary biology can help to understand interactions between holobiont members. Conversely, little is known on whether and how malignant cells are influenced by and/or exploit biological rhythms. Like many parasitic organisms, malignant cells depend on their host for sustenance, proliferation, and dispersal, and thus exploit their host for energy and resources. Therefore, although cancer cells are issued from the self (i.e., are not an exogenous organism), with an evolution that last only few decades at most (thus probably preventing the fine tuning of many adaptive responses, unlike true parasitic species where selection occurs during tens of thousands to millions of years), some adaptations that have evolved in the context of host-parasite interactions could also be relevant to cancer progression and dissemination (circulating tumor cells [CTCs]). Such adaptations might be relevant also for clinical applications, for instance in the *liquid biopsy* field (Fig. 1). Here, we combined mechanistic and evolutionary knowledge to investigate and discuss to which extent biological rhythms, especially the circadian rhythm, should be considered when studying the complexity of host-tumor cell interactions during tumorigenesis. We first provide an overview of the geological, physiological, cellular, and molecular factors implicated in circadian rhythms, then describe the impact of biological cycles on cancer development. Finally, we discuss how these principles could be implemented in novel therapeutic and preventive strategies related to the *liquid biopsy* field.

## Geological, physiological, cellular, and molecular factors of biological cycles

The movement of the Earth around the Sun and around its own axis are the main factors contributing to geophysical rhythms that result in the biological cycles observed in nature and have been a major evolutionary force. The alteration of any of these cycles may directly or indirectly cause a higher vulnerability to various infectious and/or chronic pathologies, including cancer [23–25]. All biological cycles and rhythms share similar physiological mechanisms. Nevertheless, it is possible to divide them according to the environmental factors they are associated with (e.g., sunlight, temperature, humidity, gravity, exercise, social cues, eating patterns). These cues are called “zeitgebers” or synchronizing cues, and the most predominant are those dictated by geophysical signals, such as the tidal, diel, lunar, and annual rhythms [11].

One of the most studied “zeitgebers” is the diel cycle, which is the fluctuation between day and night. The diel cycle dictates the biology of the circadian rhythm, with a duration of 24 to 26 h. Its main cue is exposure to sunlight that guides the central biological clock in vertebrates. This biological clock works as a network that is called the *vertebrate circadian axis* and includes the suprachiasmatic nucleus (SCN) in the hypothalamus, the eyes, and the pineal complex [26–28]. The relative importance of each axis component varies in different species, depending on the selective pressures in the photic niche they occupy [29]. In mammals, the SCN has the main role, and the whole cycle is abolished upon removal of this organ.

**Fig. 2:**
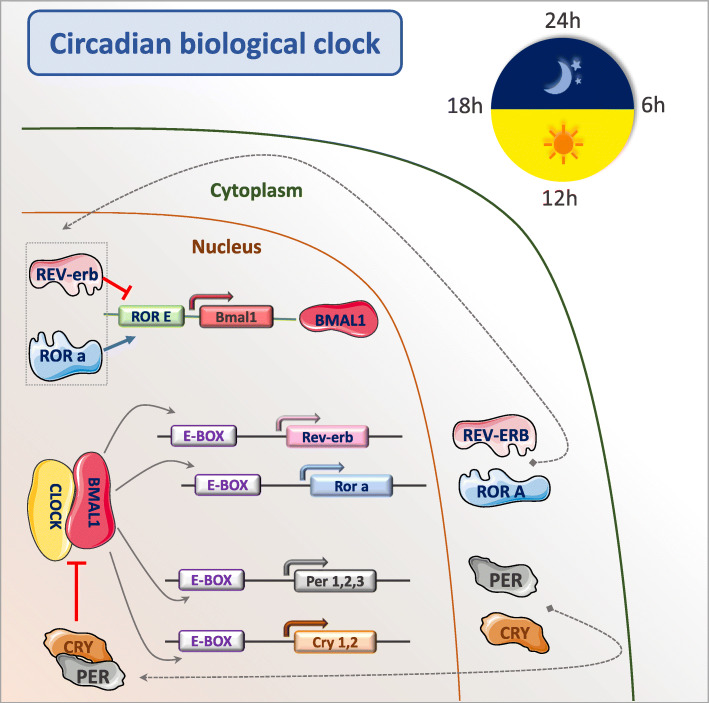
The circadian clock system is a complex transcriptional–translational autoregulatory network with activating and inhibitory components. Brain Muscle Arnt-Like protein-1 (BMAL1), the major component of the endogenous clock, heterodimerizes with Circadian Locomotor receptor Cycles Output Kaput (CLOCK) or Neuronal PAS domain protein 2 (NPAS2) to generate active transcription factor heterodimers. Binding of these dimers to the Enhancer-box (E-box) elements of their target genes leads to the expression of genes that encode the transcription repressors Cryptochrome (CRY1 and CRY2) and Period (PER1, PER2, PER3) [30, 31, 33, 34]. CRY and PER complexes inhibit CLOCK/BMAL1 transcriptional activity. CLOCK/BMAL1 dimers also drive the transcription of the nuclear receptors REV-ERBα and retinoid-related orphan receptor α (RORα) that represses and activates BMAL1 transcription, respectively [35]. Clock genes regulate the expression of clock-controlled regulators and also of genes that can be implicated in tumorigenesis. Therefore, their dysregulation might affect several cancer-related processes such as cell-cycle control, apoptosis, metabolic regulation, and DNA damage response. Circadian rhythm disruption might play a more critical role in tumor formation and progression than genetic factors [36]. Aberrant expression of circadian genes has been observed in different human cancers: head and neck squamous cell carcinoma, leukemia, ovarian, oral, and prostate cancer [37–41]

In the SCN, the circadian rhythm is regulated by molecular mechanisms that set a transcription-translation feedback loop. Indeed, cells in the SCN are synchronized and use the proteins Brain Muscle Arnt-Like protein-1 (BMAL1) and Circadian Locomotor receptor Cycles Output Kaput (CLOCK) as transcription factors to regulate the expression of several genes related to the circadian cycle [30]. BMAL1 and CLOCK also induce the expression of members of the Period (PER) and Cryptochrome (CRY) families [31, 32] that accumulate and repress the function of these two transcription factors (Fig. 2).

## Biological cycles and cancer

Biological cycles influence nearly all physiological and biological aspects of an organism, and acute and chronic alterations of the circadian rhythm have been linked to various health issues and diseases [35, 42]. Humans evolved to be active during the day and to sleep at night. Recent society changes, especially in developed countries, have important consequences on the circadian rhythm. For example, studies on sleep deprivation have shown that the disruption of the sleep pattern produces cognitive alterations and behavioral changes in the short term [43, 44], and mood disorders (e.g., depression) in the long term [45]. On the other hand, sleep deprivation has been used as antidepressant therapy [46]. Other studies found that circadian system disturbance by exogenous factors (night shift work, physiologic perturbation, and exposure to light at night) is associated with higher cancer incidence and poor prognosis. For instance, shift work is correlated with higher risk of breast, prostate, lung, and colorectal cancer [47–50]. A recent cohort study with almost 10 years of follow-up found a significant association between increasing breast cancer risk and mean hours of night work per week [51]. Moreover, the cancer risk is increased by the number of years that an individual has spent working during the night [52, 53]. However, a meta-analysis concluded that night shift, including long-term shift work, has little or no effect on breast cancer incidence [54]. These discrepancies are probably due to the many factors involved in cancer development and progression.

**Fig. 3:**
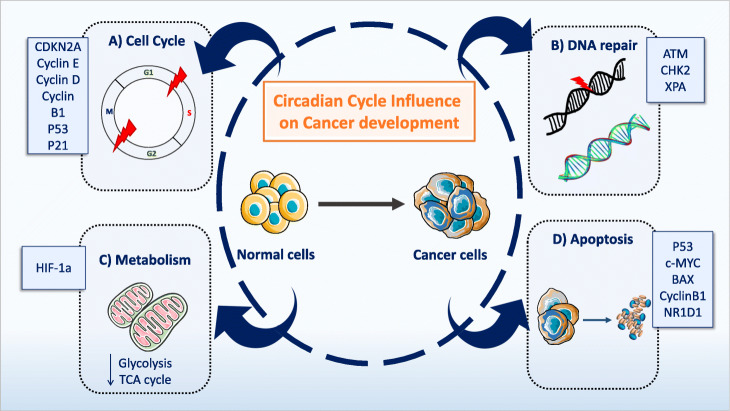
Circadian rhythms and cancer. This figure shows some examples on how circadian rhythm alterations contribute to the appearance of cancer hallmarks. **a** Circadian rhythms and cell cycle: DNA replication and cell cycle present a specific circadian pattern. Indeed, the expression of regulators of DNA replication and cell cycle shows circadian rhythms [67–70]. Moreover, circadian cycle genes play an important role in the regulation of some cell cycle genes [71, 72]. **b** Circadian rhythms and DNA repair: DNA repair, DNA damage response, and the circadian cycle are tightly connected. As observed for cell cycle regulators, the expression (mRNA and protein) of DNA repair genes shows circadian patterns [69]. Reciprocally, DNA damage can affect the circadian clock [73, 74]. **c** Circadian rhythm and metabolism: The circadian rhythm influences a wide range of metabolic processes, such as the mitochondrial, glucose, amino acid, and lipid metabolisms as well as the Krebs cycle [75, 76]. As the metabolic needs of cancer cells are different from those of normal cells, the impact of circadian disruption should be taken into account when studying their metabolism in the tumor environment. The hypoxic tumor microenvironment and the activation of hypoxia-inducible factors (HIFs) play a regulatory role in tumor-linked metabolism and angiogenesis [65, 77]. **d** Circadian rhythm and apoptosis: alterations of circadian clock components influence the expression of apoptosis-related genes

Different studies have also investigated the effect of sleep duration on cancer risk. Short sleep duration has been correlated with higher cancer risk and the development of more aggressive tumors [55–57]. Conversely, longer sleep duration reduces the risk of breast cancer [58]. Moreover, cancer recurrence has been associated with the mean hours of sleep per night before cancer diagnosis, and shorter sleep duration was correlated with higher recurrence score [59]. A study with 5 years of follow-up before and after the diagnosis of prostate cancer showed that the risk of prostate cancer is higher in men with sleep disruption [60]. However, other studies did not find any association between sleep duration or sleep disruption and cancer risk [61, 62]. Finally, circadian rhythm alterations might be an independent prognostic risk factor of poor survival in patients with cancer and of treatment side effects [63, 64]. There is strong evidence about the existence of reciprocal interactions between cancer and the circadian clock components. Indeed, alterations of the circadian regulation and homeostatic balance may facilitate the transformation of normal cells into malignant cells [35, 65]. Cancer initiation and progression are influenced by circadian cycle components through (i) direct or indirect regulation of different genes; (ii) interaction of circadian cycle proteins with other proteins; (iii) changes in redox state, cofactors, and post-translational modifications; and (iv) regulation of secreted factors with paracrine or endocrine function. These regulations have an effect on cellular processes, including nutrient metabolism, cell cycle, DNA repair, redox regulation, cellular secretion, protein folding, and autophagy [35, 66]. Figure 3 summarizes the molecular mechanisms related to the circadian cycle that are also involved in cancer development.

The circadian cycle is not the only rhythm that influences the organismal physiology. Another example in humans is the menstrual cycle that usually lasts between 24 and 38 days and is controlled by different hormones produced by the hypothalamus, pituitary, and ovaries. The risk of developing breast cancer is increased in women with higher number of cycles during their life [78, 79]. Furthermore, hormone replacement therapy and hormonal oral contraceptives promote abnormal mammary epithelial cell proliferation, resulting in higher breast cancer risk [80]. From a clinical point of view, this means that in premenopausal women, screening by mammography should be performed during the first week of the menstrual cycle [81], although this is not fully implemented in screening guidelines [82].

In addition, several studies have shown the importance of annual cycles in tumor development. For example, the seasonality of sunlight exposure and vitamin D synthesis might directly influence the risk of cancer [83–87]. Moreover, the analysis of long-term survival time series in function of the season of cancer diagnosis found a reduction in death rates among patients in whom breast cancer was diagnosed in fall [88]. Another study on skin cancer in Norway showed no significant variation of incidence and mortality rates in relation with seasons. However, a significant seasonal variation of cancer prognosis was observed [89]. Other factors might influence cancer development during the year. Indeed, skin cancers might result from excessive exposure to ultraviolet light in tropical regions [90], and warmer air temperatures have been associated with lower cancer death rates [91]. As annual cycles are hard to study and many cofounding factors might influence cancer outcome, there is still no clear evidence of their direct role in cancer.

## The influence of biological cycles on the neuroimmune-endocrine system and its clinical implications for cancer progression and dissemination

In addition to the central biological clock in the SCN, all cells and tissues have some molecular clocks. These peripheric biological clocks strongly modulate the neuroimmune-endocrine system.

The immune system is significantly influenced by the circadian cycle through various hormonal and molecular pathways, particularly the hypothalamus-pituitary-adrenal axis (HPAA), where the secretion of the glucocorticoid hormone cortisol (hydrocortisone) from the suprarenal cortex peaks early in the day [92–94]. Glucocorticoid receptors are found in the whole organism, and their main effects concern (but are not limited to) the glucose metabolism and immune system [95]. In rheumatic diseases, the use of hydrocortisone as anti-inflammatory drug increases the risk of infections [96], and the cortisol circadian peak might predispose to infections at specific times of the day. Moreover, some infectious diseases show clearly circadian patterns of symptoms. For instance, in patients with tuberculosis, fever is often exacerbated at night [97]. In infectious diseases, this difference in fever patterns might be an adaptive trait to limit the symptoms of infection to the night, thus promoting viability during the day. Additionally, it has been shown that the expression of interleukin-6 (IL-6), the main mediator of fever, can be regulated by *Mycobacterium tuberculosis* in infected macrophages [98]. This facilitates infection progression because IL-6 also stimulates the secretion of cortisol during the immune system activation [99]. Moreover, in humans, Il-6 secretion follows a circadian rhythm [100]. These circadian mechanisms might be extrapolated to cancer as well. For instance, in patients with lymphoma, cytokines are strongly released by malignant leukocytes during the night, leading to nocturnal fever peaks [101]. Moreover, in pancreatic and colorectal cancer, Il-6 has been shown to support the formation of a pro-metastatic niche in the liver [102]. Currently, it is not fully understood how cancer-induced nighttime fever may be beneficial to cancer cells from a fitness perspective; however, its presence and high Il-6 concentrations are often considered bad prognostic factors [103–106].

In a different manner, the central biological clock can influence the peripheric biological clocks not only via the HPAA, but also via the sympathetic nervous system (SNS) by using epinephrine and norepinephrine as signaling molecules. The SNS is mainly active during daytime and induces an anti-inflammatory environment, in a similar manner to glucocorticoids. The SNS might also have a role in cancer progression. Indeed, in prostate cancer, the formation of sympathetic nerve fibers contributes to cancer development and dissemination [107], and sensory nerves are induced to transdifferentiate into sympathetic nerves directly by cancer cells [108].

Both HPAA and SNS are downregulated in the early sleep period when different hormones, such as leptin, melatonin, growth hormone, and prolactin, reach their peak. These hormones have synergic pro-inflammatory actions [95]. Additionally, melatonin effectively inhibits epithelial to mesenchymal transition (EMT), which is considered one of the main mechanisms for cancer dissemination, via different pathways, such as reduction of IL-1β/NF-κB/MMP2/MMP9 signaling [109], and inhibition of Twist/Twist1 expression [110]. High melatonin levels also significantly suppress cell proliferation and induce apoptosis by inhibition of cyclooxygenase-2 and p300 [111]. Melatonin can also suppress cell invasion/migration through MMP-9-mediated ECM remodeling [112]. Melatonin also exerts inhibitory effects on metastatic HER2/neu-negative breast cancer cell migration and invasion of by repressing a panel of mesenchymal genes that regulate EMT [113]. Moreover, during the melatonin peak, hematopoietic stem cell self-renewal is enhanced through CD150 upregulation [114]. CD150 family members are key regulators of leukocyte activation and differentiation [115]. The daily cycle and secretion of different hormones are essential for the synchronized maturation of blood cells [114].

Furthermore, the immune performance of an organism is reduced by circadian cycle alterations. For example, sleep deprivation for just 24 h can drastically decrease the efficacy of the hepatitis A vaccine, an effect that is also associated with the lower cortisol release during the night [116]. This effect can be explained by the presence of two different “immune environments” in the day and in the night. Dimitrov et al. found that the number of effectors CD8^(+)^ T lymphocytes reaches its maximum peak during daytime. This outcome is driven by β-adrenergic and fractalkine (CX3CR1) receptors that are strongly expressed in these cells and increase the influence of catecholamines, related to the SNS [117]. In contrast, the number of naive T lymphocytes is lower during the day (and increases at night) when they are redistributed to the bone marrow by chemoattraction to the C-X-C motif chemokine 12 (CXCL12), the ligand of C-X-C chemokine receptor type 4 (CXCR4) that is strongly expressed on these cells. Likewise, He et al. demonstrated that CXCR4 governs the circadian fluctuation of leukocytes and myeloid lineages, such as neutrophils. Moreover, they showed that *BMAL1* gene ablation in leukocyte subsets leads to a reduction of these circadian fluctuations [118].

These observations can be interpreted as different immune reaction mechanisms during the circadian cycle, in which effector CD8^(+)^ T lymphocytes represent the immediate immune reaction during the day, and naive T lymphocytes are the main actors of the adaptive (lasting) immune reaction during the night [117]. From an evolutionary perspective, this means that during cancer cell dissemination, CTCs must adapt and elude the immediate immune response that is most active during daytime, and evade the adaptive immune response during the night. Interestingly, CXCR4 is strongly expressed in several cancer types [119, 120] and in CTCs is associated with poor prognosis [121]. Therefore, we could hypothesize that similarly to naïve T lymphocytes, CTCs might migrate to the bone marrow at specific times of the day to escape the immune system [122]. Additionally, a recent study found that CTCs are associated with neutrophils, and proposed that this might enhance their metastatic potential [123]. It might be possible that due to the neutrophil fluctuations associated with the circadian rhythms, the formation of CTC clusters is facilitated at specific times of the day, or upon disruption of the circadian cycle. Moreover, in an in vivo mice model, chronic circadian rhythm disruption enhances the CXCL12-CXCR4 axis activity and chemoattraction to component of the C-X-C motif chemokine 5-C-X-C chemokine receptor type 2 (CXCL5-CXCR2) axis. These two axes recruit myeloid-derived suppressor cells into the tumor micro-environment, promoting cancer cell dissemination and metastasis formation. In agreement, inhibition of the CXCL5-CXCR2 axis limits the number of metastasis in mice [124]. The higher expression of components of these two axes upon circadian disruption might increase CTC fitness.

On the other hand, expression of specific markers can give some advantages to CTC subsets that will be selected. One example is the acquisition by CTCs of the “perfect” phenotype to escape the immune system. Indeed, expression of PD-L1 (*a camouflage to trap the immune system*) and/or CD47 (*the “do not eat me” signal*) by CTCs certainly help these disseminating tumor cells to survive the attack by immune cells. A recent study in *Bmal1*^−/−^ mice showed that *Bmal1* (one of the two main regulators of the circadian cycle) counter-balances *Pd-l1* expression in macrophages and plays a role in the immune response [125]. This kind of mechanism could, in theory, also be involved in CTC clearance from blood during the day, with possible implications for the sampling time and evaluation of this marker in CTCs.

Another interesting observation, with potential consequences for tumor dissemination, is that circadian variations can predispose to higher platelet activation and coagulation, thus potentially promoting CTC survival. For instance, cardiovascular events have day/night patterns with peaks in the morning, suggesting a potential link with the endogenous circadian clock that controls platelet activation. This hypothesis is supported by the observation that the expression peaks of platelet surface activated glycoprotein (GP) IIb-IIIa, GPIb, and P-selectin also occur at that time [126]. These molecules are involved in the reciprocal interaction of platelets and CTCs. Indeed, platelet P-selectin interacts with tumor cell CD44, and the fibrinogen receptors GPIIb-IIIa are involved in platelet adherence on CTCs and in the formation of platelet–CTC emboli [127]. Moreover, platelets can protect CTCs by forming a protective cloak and by conferring a “pseudo-normal” phenotype against natural killer cells [128]. As platelet-coated CTCs have been detected in the blood of patients with metastatic cancer [129], the circadian peaks of platelet activation and of surface marker expression suggest possible roles for the circadian clock in CTC escape from the immune system. However, the role of the circadian cycle in CTCs and the potential consequences on CTC clone fitness have not been elucidated yet.

These observations might have implications for CTC use as *liquid biopsy*. Most studies on CTCs do not report the sampling time [130, 131]. Only one group tried to determine the circadian cycle role in tumor cell dissemination. To evaluate a possible circadian variation in CTC number in patients with metastatic breast cancer, they carried out two studies (*n* = 51 and *n* = 23 samples) in which CTCs were quantified 12 h apart (at 8:00 a.m. and at 8:00 p.m. of the same day) [130, 131] The authors concluded that CTC count, determined with the CellSearch® system, does not significantly fluctuate depending on the sampling time. These data suggest that the circadian rhythm does not influence tumor cell dissemination; however, they need to be confirmed in a bigger cohort of patients in a multi-center trial involving independent research groups. Moreover, blood samples were not collected during the night. Additionally, the Nyquist theorem states that to detect a cycle, sampling rate must be at least twice the signal frequency [132, 133]. Two samples may theoretically be enough to show crude day/night differences, but the cycle amplitude (e.g., expressed in number of CTCs) may be difficult to estimate, if samples are not collected when CTC numbers are highest and lowest. Therefore, at least four samples equidistant in time should be required to clearly define a cycle. Moreover, only two data points for 24 h might not allow highlighting possible scenarios in which CTC number increases during a short time window. These statements need to be considered in the design of future studies.

**Fig. 4:**
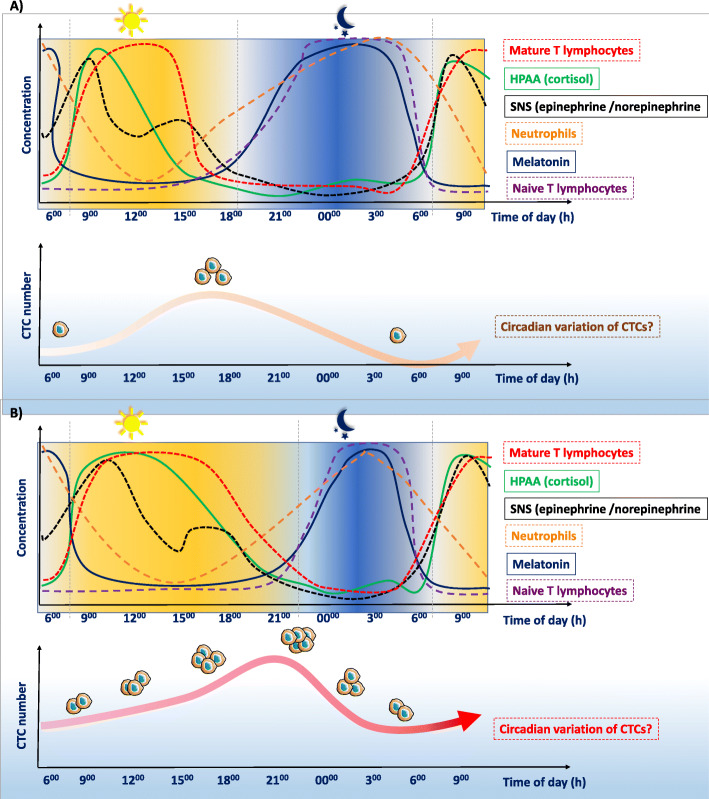
Circadian cycle influence on cancer cell dissemination. The circadian rhythm influences the neuroimmune-endocrine system, with clear differences between day and night. These observations can be summarized as two synergic “immune environments”. Disruptions of these biological cycles can facilitate cancer progression and dissemination of circulating tumor cells (CTCs). Therefore, cancer cells must adapt to efficiently progress through the metastatic cascade. Potential influence of the **a** normal and **b** disrupted circadian cycle on CTCs and cancer dissemination

Furthermore, independently of CTC number in blood, the biology of each CTC can be different according to its neuroimmune-endocrine environment. For instance, expression of CXCR4 might fluctuate in CTCs during the day, and this might have important clinical implications. Indeed, these tumor cells can migrate and hide in specific niches in the bone marrow, which is considered a “reservoir” for disseminated cells where they can enter dormancy after having left the primary tumor and survived in the circulation [20]. In addition, CTCs might take advantage of the anti-inflammatory effects of cortisol (the secretion of which is strongly increased upon sleep disruptions) to escape the immune surveillance and survive. Moreover, alterations in the circadian cycle will modify the influence of this cycle on cancer cell dissemination (Fig. 4). Altogether, the possible fluctuations related to the biological cycles may play a crucial role in defining the best moment for blood sampling to increase the chance of CTC detection.

On the other hand, the knowledge on circadian biology has been successfully applied to maximize the balance between chemotherapy efficacy and toxicity. For instance, the early clinical trials on oxaliplatin, which is one of the main therapies against colorectal cancer, showed high toxicity, and therefore, its further development was stopped [134]. However, a chronopharmacology approach allowed identifying the best time for oxaliplatin administration, strongly improving patient survival [135]. In the *liquid biopsy* field, the circadian cycle-based schedules have been rarely implemented, although it would be important to include this strategy in future clinical trials on *liquid biopsy* and in cancer research in general [136].

## Evolutionary considerations

The data described in the previous sections indicate that biological rhythms, especially the circadian cycle, should not be neglected when studying cancer. The circadian rhythm influences the host defense efficiency against cancer. Moreover, malignant cells, like any other cell in the holobiont, are directly or indirectly influenced by the host internal rhythms.

In addition, it should be important to determine whether during tumorigenesis, malignant cells can acquire some features, like parasites and microbiota, that allow them to exploit their host circadian rhythm, and/or even to manipulate it to improve their proliferation and dispersal. One major difference between parasitic organisms/bacteria and malignant cells is that in most cases, cancer cells are not transmitted between hosts (but see [137]). Thus, although cancer is an ancestral disease [138], each cancer must “reinvent the wheel” because their evolutionary adaptations disappear when their host dies. As the “lifestyle” of malignant cells is under selective pressure for only few decades, highly sophisticated adaptive responses, as observed in organisms exposed to natural selection for millions of years, may be unlikely to appear [139]. On the other hand, in only few years, natural selection can act on the extreme diversity of cells typically generated by tumors, leading to the emergence of clones that show the best features for malignant progression, metastasis development, and resistance to immune attack and to therapies. In such “successful” tumors, adaptations in relationship with the host circadian rhythm may be detected.

Assuming that the conditions allowing the selection of these adaptive traits are present, two hypotheses concerning the behavior of malignant cells, especially CTCs, relative to the host circadian rhythm can be proposed. First, oncogenic selection might lead to the emergence of malignant cells that avoid, or limit their activities during the part of the circadian rhythm that is not favorable or dangerous for them, and amplify them during advantageous time windows (see for instance [140, 141]). Second, malignant cells could acquire the ability to alter their host circadian cycle to promote directly or indirectly their proliferation or dispersal (see for instance [142]).

Concerning the first hypothesis, the number of circulating immune cells in humans varies during the circadian cycle [118, 143]. Moreover, some studies highlighted the asynchrony in cell proliferation and metabolism between host and malignant tissues [144]. Finally, there is experimental evidence that some periods of the day are better than others for cancer cell proliferation and spread. For instance, using two genetically distinct mouse strains and two different tumor cell models (i.e., fibrosarcoma and melanoma), Hrushesky et al. [145] determined the tumor-take frequency after subcutaneous tumor cell inoculation, and the number of pulmonary tumor nodules after intravenous tumor cell injection at six equidistant times of the day. With fibrosarcoma cells, they found that tumor-take frequency was lower after injection close to the daily sleep/wake boundary. Similarly, with melanoma cells, the daily sleep/wake boundary was the time associated with the greatest resistance to metastatic spread. In this context, it is not surprising that therapies altering the tumor biological rhythms are increasingly considered as efficient to prevent cancer proliferation and spread, in addition to their limited toxicity for the surrounding healthy tissue [146].

Concerning the second hypothesis, few studies found that in patients with cancer (notably breast and ovarian cancer [147, 148]), the distinction between nighttime and daytime activities is reduced, suggesting a possible circadian rhythm disruption. Insomnia is also frequently reported by patients with breast, gynecological, and lung cancer [149]. Although these circadian rhythm disorders may have a variety of causes, a direct effect of the tumor on sleep is possible [149], for instance by affecting the secretion of the cytokines that modulate the sleep-wake cycle. Interestingly, several studies have suggested that the aberrant clock gene expression observed in many tumors might promote cancer cell survival, and have identified the mechanisms through which malignant cells induce sleep disruption (see [150] for a recent review). However, they did not consider their findings in the framework of a potential adaptive context of host manipulation. We strongly believe that the hypothesis of circadian rhythm manipulation by cancer is an additional reason to adopt a Darwinian approach in cancer research. More studies are needed to determine whether cancer cells with the ability to disrupt circadian rhythms are directly favored by selection, or whether this is only a side effect with positive effects on carcinogenesis. In any case, in accordance with the idea that fighting cancer adaptation might improve therapies, pharmacological modulation of clock-related proteins (REV-ERB receptor activity) is increasingly considered as an effective anticancer strategy [151].

## Concluding remarks

While the circadian timing of cancer treatments is progressively acknowledged, e.g., [152–154], few studies have tested the hypothesis that malignant cells could exploit and/or manipulate the host biological rhythms. Yet, it is important to determine whether, when, and how malignant cells can acquire such adaptive traits in order to prevent their selection and to limit their spreading. This is particularly true for CTCs because this could strongly influence the speed and rate of expansion of invasive cancers. As several non-genetic variables are involved in tumor formation and progression, it is also essential to determine whether the acquisition of the ability to exploit/manipulate circadian rhythms by malignant cells has to be considered within the framework of phenotypic plasticity or just as a genetic adaptation. It is also important to consider the whole holobiont in which these malignant adaptations could occur, by considering the microbiota and the parasitic organisms present in the host, because they may have common or conflicting interests concerning the host exploitation, e.g., [155]. Only such an integrative approach will provide a correct assessment of the context in which malignant cells that can exploit/manipulate the host biological rhythms are selected. As one single discipline or biological model cannot correctly describe all these phenomena, scientists from different fields must engage in exchanges and collaborations. Demonstrating that malignant cells can exploit/manipulate their host biological rhythms is only the first step. Indeed, this knowledge will have to be integrated in the design of preventive or curative strategies, and for improving the identification of life periods when the risk of invasive cancer initiation is highest. It will also be necessary to determine whether these adaptations rely on a more or less constant/obligatory sequence of events, with the same proximate factors (i.e., evolutionary convergence) that could potentially be targeted by specific therapies. Another promising research direction is to explore the proximate mechanisms used by parasites that also manipulate the host circadian rhythms to favor their own fitness, e.g., [156]. If such strategies rely on similar biochemical precursors, it would be important to test whether therapies that target manipulative activities in parasites might be equally effective against manipulation by cancer cells (see for instance [157]).

## Supplementary Information

**Additional file 1.** Review history.

## Data Availability

Not applicable.
