# Supplementary material for: Do malignant cells sleep at night?
Source: Genome Biol. 2020 Nov 12;21:276. doi: 10.1186/s13059-020-02179-w (PMC7659113; doi:10.1186/s13059-020-02179-w)
Supplement: Supplementary file 1 — Additional file 1. Review history. [file 13059_2020_2179_MOESM1_ESM.docx]

Review History

**First round of review**

**Reviewer 1**

**Comments to author:**

This is a very interesting and original review on the influence of biological rhythms on the metastatic process. Objective data are limited but this comprehensive review is useful generating new hypothesis in this field.

A word a caution in the conclusion could be recommended in the conclusion as the analogy between cancer and parasite is limited by the fact that cancer cell are issue of the self and are not an exogenous organism

Minor comment: presentation of several references should be corrected ex: ref 36, 88, 89 etc.

**Reviewer 2**

**Comments to author:**

Cortés-Hernández and Eslami-S et al. reviewed and discussed the extent to which biological rhythms (circadian rhythms) impact and/or are affected during tumorigenesis via host-tumour interactions. The review provides a brief overview of the circadian clockwork in vertebrates, its role in cancer progression and emphasizes on the host-tumour interplay from an evolutionary perspective and the opportunities/possibilities for cancer cells to benefit from and manipulate the circadian clock of the host.

Overall, the review contains relevant publications for the different sections mentioned. The text is well written and easy to follow. The evolutionary aspect of circadian host-tumour interactions is particularly interesting, as well as the comparison of cancer cells inside the host to parasitic organisms/bacteria.

While I mostly appreciated the work here presented and recognize its interest, I have a few comments which should be addressed before publication.

1-	Clinical implications and the example of liquid biopsy are not fully covered. There are methods/publications suggesting how chrono modulated therapies can affect patient outcome, which are not mentioned. More literature data could have been provided here (e.g. see below publications).

2-	Generally, the publications listed are not very recent. More recent review articles are relevant for the topics addressed and should, in my opinion, be cited: Sulli et al. (2018), Cederroth et al. (2019), Rijo-Ferreira &. Takahashi (2019), which discuss the role of biological rhythms in health and disease, chronotherapy as well as host-pathogen interactions and (Basti et al. 2020), which highlights the putative interaction between host-tumour cells with impact on the circadian clock.

3-	In the first section of the manuscript" Geological, physiological, cellular and molecular factors of biological cycles" the authors introduced the term "Zeitgeber" limited to to physical and geographical cues while the actual term has a broader meaning including for e.g. Social cues, Exercise, Eating/drinking.

4-	Minor: p. 8, line 4: sentence is not complete.

5-	Minor: p.8, 1st paragraph: reference is missing.

6-	Minor: I recommend to use the 24-hour time, instead of the 12-hour time in the figure. In the current version of the figure 1, 24h is represented as 12pm (should be 12 am) and 12h is represented as 12 am (should be 12pm).

**Reviewer 3**

This article is rather vague and not comprehensive enough especially with the circadian clock and its role in cancer. Authors ignored a lot of literature with circadian clock connection on hallmarks of cancer especially with DNA repair, cell cycle, and metabolism. The circadian clock connection with cancer both at mechanistic and epidemiological level is sufficient. I suggest that authors need to bring a renowned circadian biologist to this review as one of the authors if they wanted to publish in high impacts journals like Genome Biology.

**Comments to author:**

**Authors Response**

**Point-by-point responses to the reviewers’ comments:**

**Reviewer #1**:

This is a very interesting and original review on the influence of biological rhythms on the metastatic process. Objective data are limited but this comprehensive review is useful generating new hypothesis in this field.

We would like to thank reviewer 1 for her/his positive impression regarding our manuscript. We have revised the manuscript according to her/his comments and modified it point-by-point. All modifications can be found in the manuscript highlighted in yellow.

A word a caution in the conclusion could be recommended in the conclusion as the analogy between cancer and parasite is limited by the fact that cancer cell are issue of the self and are not an exogenous organism.

**RE:** We agree with this observation and clarified this specific point in the *Introduction* (page 5).

Minor comment: presentation of several references should be corrected ex: ref 36, 88, 89 etc.

**RE:** All references have been corrected.

**Reviewer #2:**

Cortés-Hernández and Eslami-S et al. reviewed and discussed the extent to which biological rhythms (circadian rhythms) impact and/or are affected during tumorigenesis via host-tumour interactions. The review provides a brief overview of the circadian clockwork in vertebrates, its role in cancer progression and emphasizes on the host-tumour interplay from an evolutionary perspective and the opportunities/possibilities for cancer cells to benefit from and manipulate the circadian clock of the host.

Overall, the review contains relevant publications for the different sections mentioned. The text is well written and easy to follow. The evolutionary aspect of circadian host-tumour interactions is particularly interesting, as well as the comparison of cancer cells inside the host to parasitic organisms/bacteria.

While I mostly appreciated the work here presented and recognize its interest, I have a few comments which should be addressed before publication.

**RE:** We would like to thank reviewer 2 for the time that she/he spent reading our manuscript; her/his insightful and very constructive comments as well as suggestions helped to improve it. We have revised the manuscript according to her/his comments and modified it point-by-point. All modifications can be found in the manuscript highlighted in yellow.

1- Clinical implications and the example of liquid biopsy are not fully covered. There are methods/publications suggesting how chrono modulated therapies can affect patient outcome, which are not mentioned. More literature data could have been provided here (e.g. see below publications).

**RE:** We added a new box with more information on *liquid biopsy* (**Box 1**). We also followed the reviewer’s recommendations and added a paragraph on the use of chronopharmacology for oxaliplatin, and how this could potentially be applied in the *liquid biopsy* field (page 16, at the end of the section “The influence of biological cycles on the neuroimmune-endocrine system and its clinical implications for cancer progression and dissemination”).

2- Generally, the publications listed are not very recent. More recent review articles are relevant for the topics addressed and should, in my opinion, be cited: Sulli et al. (2018), Cederroth et al. (2019), Rijo-Ferreira &. Takahashi (2019), which discuss the role of biological rhythms in health and disease, chronotherapy as well as host-pathogen interactions and (Basti et al. 2020), which highlights the putative interaction between host-tumour cells with impact on the circadian clock.

**RE:** We added additional information from these reviews in **Box 2** and in the main text; we also quoted these reviews when needed.

3- In the first section of the manuscript" Geological, physiological, cellular and molecular factors of biological cycles" the authors introduced the term "Zeitgeber" limited to physical and geographical cues while the actual term has a broader meaning including for e.g. Social cues, Exercise, Eating/drinking.

**RE:** Modification done.

4- Minor: p. 8, line 4: sentence is not complete.

**RE:** Modification done.

5- Minor: p.8, 1st paragraph: reference is missing.

**RE:** Modification done.

6- Minor: I recommend to use the 24-hour time, instead of the 12-hour time in the figure. In the current version of the figure 1, 24h is represented as 12pm (should be 12 am) and 12h is represented as 12 am (should be 12pm).

**RE:** Modification done.

**Reviewer #3:**

This article is rather vague and not comprehensive enough especially with the circadian clock and its role in cancer. Authors ignored a lot of literature with circadian clock connection on hallmarks of cancer especially with DNA repair, cell cycle, and metabolism. The circadian clock connection with cancer both at mechanistic and epidemiological level is sufficient. I suggest that authors need to bring a renowned circadian biologist to this review as one of the authors if they wanted to publish in high impact journals like Genome Biology.

**RE:** We would like to thank reviewer 3 for the time that she/he spent reading our manuscript to improve it. We took her/his comments into consideration to revise our manuscript.

We have improved several ideas through the main text to support the points presented. Moreover, we were aware of the extensive literature about ‘circadian cycle’ and ‘cancer’; however, in our manuscript, we wanted to describe the specific circadian influence on the physiological systems that increase tumor cell fitness for tumor dissemination, from an evolutionary point of view and how this might influence the detection of circulating tumor cells as liquid biopsy of solid cancer. We significantly improved and modified **Box 2** to include examples from the literature about other cancer hallmarks showing circadian influence: DNA repair, cell cycle, and metabolism in cancer. All modifications can be found highlighted in yellow.

**Second round of review**

**Reviewer 2**

The authors have addressed my concerns and I find the manuscript to be much improved.

I only have one minor comment. In figure 2, it is not indicated the units of time, it is better to write in the X-axis "Time of day [h]"
